# Yeast-Based Biosynthesis of Natural Products From Xylose

**DOI:** 10.3389/fbioe.2021.634919

**Published:** 2021-02-03

**Authors:** Jian Zha, Miaomiao Yuwen, Weidong Qian, Xia Wu

**Affiliations:** School of Food and Biological Engineering, Shaanxi University of Science and Technology, Xi’an, China

**Keywords:** xylose, yeast, natural product, *Saccharomyces cerevisiae*, *Pichia stipitis*, *Yarrowia lipolytica*

## Abstract

Xylose is the second most abundant sugar in lignocellulosic hydrolysates. Transformation of xylose into valuable chemicals, such as plant natural products, is a feasible and sustainable route to industrializing biorefinery of biomass materials. Yeast strains, including *Saccharomyces cerevisiae*, *Scheffersomyces stipitis*, and *Yarrowia lipolytica*, display some paramount advantages in expressing heterologous enzymes and pathways from various sources and have been engineered extensively to produce natural products. In this review, we summarize the advances in the development of metabolically engineered yeasts to produce natural products from xylose, including aromatics, terpenoids, and flavonoids. The state-of-the-art metabolic engineering strategies and representative examples are reviewed. Future challenges and perspectives are also discussed on yeast engineering for commercial production of natural products using xylose as feedstocks.

## Introduction

Biomass hydrolysates are frequently used as a feedstock in biomass-based biorefinery. The conversion of these hydrolysates to useful compounds, such as natural products, is usually carried out by yeasts due to their advantageous properties such as tolerance of toxic inhibitors in hydrolysates, functional expression of eukaryote-derived heterologous pathways, and resistance to osmotic stress and harmful fermentation stimuli (Kwak et al., 2019; Li et al., 2019). A major challenge in this process is a waste of resources and high process cost partly due to inefficient utilization of xylose, which is the second most abundant saccharide in biomass hydrolysates (Zha et al., 2012, 2014). Xylose cannot be naturally metabolized by the commonly used yeast chassis such as *Saccharomyces cerevisiae* and *Yarrowia lipolytica* with the exception of *Scheffersomyces stipitis* (Jagtap and Rao, 2018). To overcome this difficulty, there have been many attempts over the past few decades on yeast engineering for xylose fermentation, with *Sa. cerevisiae* as the main focus (Hou et al., 2017).

The early endeavor of yeast engineering largely aimed at bioethanol production using *Sa. cerevisiae* and *Sc. stipitis* due to their intrinsic capability of synthesizing ethanol from various carbon sources. However, the complicated production process, including pretreatment, saccharification, and fermentation, results in high production cost and limited market competitiveness, and the relevant technologies are far from industrialization on a large scale (Kwak et al., 2019). In consequence, researchers started to explore yeast-based conversion of xylose, together with glucose, into value-added chemicals that are not readily available through extraction or chemical synthesis (Kwak and Jin, 2017; Kwak et al., 2019).

Although glucose is a preferred carbon source for many microbes, it is not always better than xylose when particular metabolic requirements need to be met. Xylose can induce respiratory effects on central carbon metabolism even under anaerobic conditions, and the metabolic flux of the TCA cycle, the pentose phosphate pathway (PPP), and acetyl-CoA biosynthesis is much higher on xylose than on glucose (Kwak et al., 2019). Compared with glucose, xylose induces a different metabolic flux distribution and enhances the generation of some key intermediate metabolites such as acetyl-CoA, malonyl-CoA, and erythrose-4-phosphate (E-4-P; Kwak and Jin, 2017). The elevated supply of these precursors is beneficial for the production of several classes of natural compounds such as terpenoids and aromatics.

In this review, we will summarize the recent progress on genetic modification of yeast strains for the biosynthesis of natural products using xylose as the carbon source, with a focus on three yeasts including the natural xylose-fermenting yeast *Sc. stipitis* and recombinant xylose-fermenting yeasts *Sa. cerevisiae* and *Y. lipolytica*. For each of these organisms, the description will be mainly centered on four aspects, which include general physiology related to metabolic properties, tools, and strategies available for genetic manipulation, metabolism of xylose, and the biosynthesis of typical natural products from xylose.

## Xylose Metabolic Pathways

**Figure 1:**
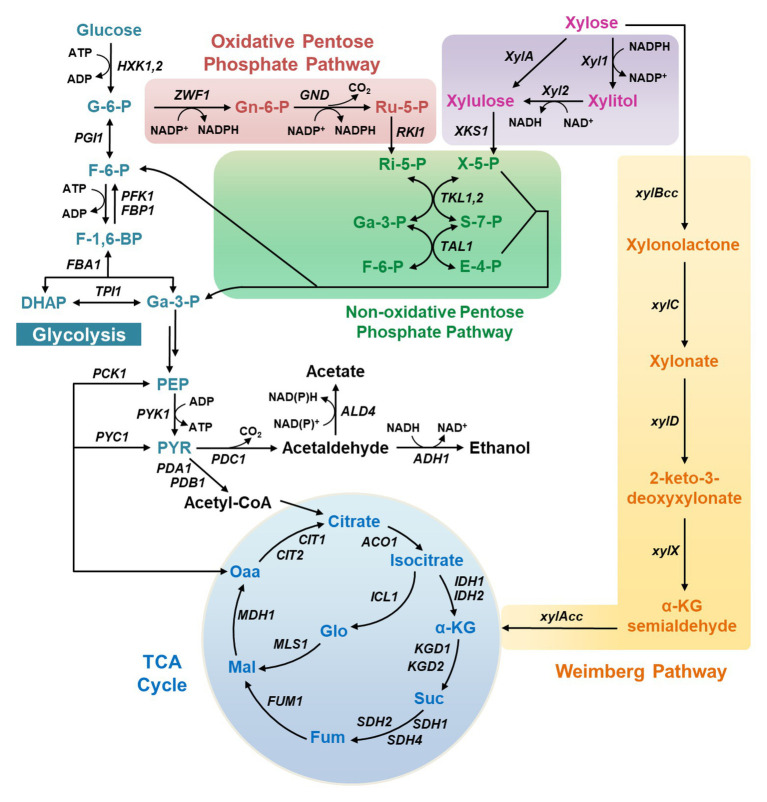
The metabolic pathways for xylose metabolism in yeasts. X-5-P, xylulose-5-phosphate; Ri-5-P, ribose-5-phosphate; Ru-5-P, ribulose-5-phosphate; Ga-3-P, glyceraldehyde-3-phosphate; S-7-P, sedoheptulose-7-phosphate; F-6-P, fructose-6-phosphate; E-4-P, erythrose-4-phosphate; G-6-P, glucose-6-phosphate; Gn-6-P, 6-phosphogluconate; F-1,6-BP, fructose-1,6-bisphosphate; DHAP, dihydroxyacetone phosphate; Gly-1,3-P, 1,3-bisphosphoglycerate; PEP, phosphoenolpyruvate; PYR, pyruvate; Oaa, oxaloacetate; Mal, malate; Fum, fumarate; Suc, succinate; α-KG, α-ketoglutarate.

Three xylose catabolic pathways have been discovered so far in natural xylose-utilizing microorganisms (Figure 1). The first pathway is the XR-XDH pathway widely present in natural xylose-utilizing yeasts, such as *Sc. stipitis* and *Candida shehatae*, in which xylose is reduced to xylitol by xylose reductase (XR) and then oxidized to xylulose by xylitol dehydrogenase (XDH; Figure 1).

A more direct pathway is the XI pathway that converts xylose into xylulose in a single step using xylose isomerase (XI; Figure 1), after which xylulose can be channeled into glycolysis *via* phosphorylation and multiple biochemical reactions in the non-oxidative PPP. The XI pathway is intrinsic in bacteria and a few fungi.

The third xylose metabolic pathway is the Weimberg pathway, in which xylose is oxidized by xylose dehydrogenase (XylB) to xylono-γ-lactone and converted to xylonate by xylono-γ-lactone lactonase (XylC; Figure 1). Xylonate then undergoes two successive dehydration reactions by xylonate dehydratase (XylD) and 2-keto-3-deoxy-xylonate dehydratase (XylX) to form α-ketoglutarate semialdehyde, which is further oxidized to α-ketoglutarate by α-ketoglutarate semialdehyde dehydrogenase (XylA) and enters the TCA cycle.

Among these pathways, the XR-XDH route is the mostly explored in recombinant yeasts owing to its ease of expression and high metabolic flux, and this route has been successfully expressed in non-xylose-fermenting yeasts including *Sa. cerevisiae* and *Y. lipolytica* (Kim et al., 2013; Wu et al., 2019). The functional expression of bacterial XI pathway in non-xylose-fermenting yeasts is a challenge, whereas the XI pathway derived from *Piromyces* and other organisms operates well in *Sa. cerevisiae* (Kuyper et al., 2003). In comparison, the introduction of the Weimberg pathway is much more challenging, which has only been achieved recently in *Sa. cerevisiae* with albeit low efficiency of xylose metabolism (Borgström et al., 2019). In this work, the gene *xylA* was replaced with its ortholog *KsaD* from *Corynebacterium glutamicum*, and gene expression in the lower Weimberg pathway (*XylD*, *XylX*, and *KsaD*) was enhanced. Through further deletion of the iron regulon repressor *FRA2* and serial adaptive evolution, the engineered strain was capable of metabolizing up to 57% of the carbon from assimilated xylose into biomass and carbon dioxide in the mixture of glucose and xylose. The growth using xylose as the sole carbon source has not been reported, possibly due to the weak activity of the introduced Weimberg pathway.

## Biosynthesis of Natural Products From Xylose by *Scheffersomyces Stipitis*

*Scheffersomyces stipitis*, previously known as *Pichia stipitis*, is a facultatively anaerobic yeast that exists mostly in haploid form (Jeffries et al., 2007). This microbe can metabolize many lignocellulose-derived saccharides including hexoses, pentoses, and cellobiose attributed to various hydrolases encoded by its genome such as β-glucosidases, endoglucanases, xylanase, mannanase, and chitinase. Such a high diversity of carbon sources for this yeast may be a result of long adaptation to its natural habitat, as several known *Sc. stipitis* strains, such as CBS 6054, naturally dwell in insects that feed on lignocellulose. This unique capability makes *Sc. stipitis*, a promising strain to utilize lignocellulosic biomass as feedstocks, to produce biofuels and chemicals (Gao et al., 2017).

*Scheffersomyces stipitis* is a Crabtree negative yeast with greater respiratory capacity than *Sa. cerevisiae* due to the presence of an alternative respiration system beyond cytochrome system that is sensitive to salicylhydroxamic acid, the so-called SHAM-sensitive respiratory pathway (Jeppsson et al., 1995; Jeffries et al., 2007). This pathway branches out of the cytochrome pathway at ubiquinone, donating electrons directly to O_2_ to form water (Jeppsson et al., 1995). *Scheffersomyces stipitis* cells growing without a functional cytochrome pathway can still metabolize xylose although the growth rate is reduced by half (Shi et al., 1999). In addition, this yeast contains NADH dehydrogenase complex I, which is absent in *Sa. cerevisiae*, for ATP generation through oxidative phosphorylation (Jeffries et al., 2007). *Scheffersomyces stipitis* is more stable than *Sa. cerevisiae* in terms of metabolite accumulation in response to oxygen supply, and its metabolic flux distribution is greatly affected by oxygen levels, which is different from *Sa. cerevisiae* that significantly relies on glucose concentrations for metabolic regulation.

*Scheffersomyces stipitis* is a natural xylose utilizer that harbors the XR/XDH pathway. XR uses either NADPH or NADH as cofactor, and excess NADH is produced when cells are grown on xylose, allowing for oxygen-independent utilization of xylose (Bruinenberg et al., 1984; Does and Bisson, 1989; Jeffries et al., 2007). The *Sc. stipitis* genome encodes multiple enzymes to participate in NAD(P)H oxidoreductase reactions for balancing of cofactors and redox potential (Jeffries et al., 2007). This organism has the highest native capability of xylose fermentation among all the known microbes, and its rate of xylose uptake and cell growth, when xylose is used as the sole carbon source, is one order of magnitude higher than that of the recombinant *Sa. cerevisiae* harboring the XR/XDH pathway (Jeffries et al., 2007; Feng and Zhao, 2013). In *Sc. stipitis*, xylose uptake is a rate-limiting step in xylose metabolism under aerobic conditions, whereas xylulose formation from xylitol is rate-limiting under anaerobic conditions (Ligthelm et al., 1988). There are two types of xylose transporters termed the high- and low-affinity transport systems, both of which are proton symports and can be inhibited by dinitrophenol, indicating active transport (Does and Bisson, 1989). The uptake of xylose can be inhibited by glucose competitively and non-competitively in the low-affinity and high-affinity transport systems, respectively (Kilian and van Uden, 1988; Jeffries et al., 2007).

*Scheffersomyces stipitis* uses CUG to encode serine instead of leucine, which makes codon optimization extremely important when heterologous genes contain leucine-encoding CTG. In addition, nonhomologous end joining (NHEJ) dominates DNA repair of double strand breaks, leading to very low efficiency (<10%) of genome editing (Cao et al., 2018). NHEJ in this organism relies on the dimeric protein complex Ku consisting of Ku70 and Ku80 subunits that binds to ends of DNA double strand breaks. Deletion of the Ku complex increases the efficiency of homologous recombination to ~70%. Using such a Ku^−^ background, CRISPR-Cas9 and CRISPR-dCas9 systems have been developed in *Sc. stipitis*, which has greatly facilitated the basic genetic engineering. Moreover, a wide spectrum of native promoters and terminators has been reported, and a centromeric DNA sequence has been identified to stabilize episomal plasmids for stable expression of heterologous genes (Cao et al., 2017). These findings and tools make genetic engineering precise and facile in *Sc. stipitis*.

So far, *Sc. stipitis* is not as a popular host as *Sa. cerevisiae* in the production of natural products. This is partly due to the limited genetic tools such as expression plasmids and gene knock-out tools. On the other hand, the physiology and genetics of *Sc. stipitis* are less defined compared with *Sa. cerevisiae*. Nevertheless, *Sc. stipitis* has high metabolic flux toward PPP and abundant supply of E-4-P (Jeffries et al., 2007), which is the precursor of aromatics, therefore, this yeast is suitable for the biosynthesis of aromatics (Gao et al., 2017; Cao et al., 2018). Recently, *Sc. stipitis* was used to synthesize shikimate from xylose (Gao et al., 2017). In this strain, overexpression was performed on *aro4*_K220L_, *tkt1*, and *aro1*_D900A_, which encode a feedback insensitive DAHP synthase, a transketolase, and a pentafunctional enzyme converting DAHP to 5-enolpyruvyl-3-shikimate phosphate, respectively. Additionally, promoters and terminators were optimized to ensure strong constitutive expression of the pathway genes. These strategies allowed for the production of 3.11 g/L shikimate from xylose under aerobic conditions, which was 7-fold higher than the highest reported titer ever achieved in *Sa. cerevisiae*. This work opens the door to the biosynthesis of aromatic compounds in *Sc. stipitis*.

## Using *Saccharomyces Cerevisiae* as the Host to Produce Natural Products From Xylose

*Saccharomyces cerevisiae* is one of the mostly used model hosts in metabolic engineering. It is a single-celled GRAS (generally regarded as safe) fungus that proliferates through budding or fission. *Saccharomyces cerevisiae* has high tolerance to environmental stress, such as low pH, high osmotic pressure, and phage infection, making it advantageous in industrial fermentation. Moreover, the well-defined physiological information and sophisticated metabolic engineering tools of this yeast greatly facilitate its engineering and applications.

Natural *Sa. cerevisiae* contains native xylose metabolic pathway genes, such as aldose reductase-encoding *GRE3*, sorbitol dehydrogenase-encoding *SOR1* and xylulose kinase (XKS) gene *XKS1*, the counterpart of *XYL1*, *XYL2*, and *XYL3* from *Sc. stipitis* (Konishi et al., 2015). However, these genes are not expressed sufficiently to support significant growth on xylose. Metabolic engineering efforts have created excellent xylose-utilizing recombinant *Sa. cerevisiae* through various approaches including the introduction of efficient heterologous xylose metabolic pathway, activation of endogenous genes involved in xylose utilization, enhancement of the PPP, balance of cofactors, evolutionary engineering using xylose as the sole carbon source, and so on (Hou et al., 2017). These attempts have potentiated *Sa. cerevisiae* as a robust chassis organism in generating a wide variety of useful compounds using xylose alone or together with glucose as the carbon source.

**Figure 2:**
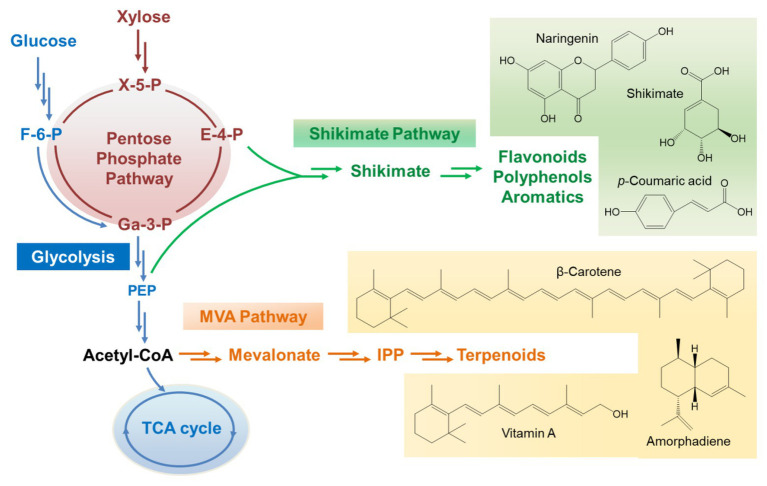
The metabolic flux channel to direct the production of natural products through the shikimate pathway and mevalonate pathway when xylose is employed as the carbon source. X-5-P, xylulose-5-phosphate; E-4-P, erythrose-4-phosphate; F-6-P, fructose-6-phosphate; Ga-3-P, glyceraldehyde-3-phosphate; PEP, phosphoenolpyruvate; IPP, isopentenyl diphosphate.

Unlike *Sc. stipitis*, *Sa. cerevisiae* is a Crabtree-positive strain. The metabolic pattern on xylose is quite different from that on glucose. The efficiency of xylose assimilation and metabolism in recombinant *Sa. cerevisiae* is generally much lower than glucose although xylose uptake and utilization has been engineered extensively and improved dramatically (Qi et al., 2015; Hou et al., 2017). Inefficient xylose assimilation leads to carbon starvation-like metabolomic patterns of glycolysis, indicated by the observation of low pools of glycolytic intermediates except for the accumulation of phosphoenolpyruvate, which is required for the production of aromatic compounds through the shikimate pathway (Mert et al., 2017). Growth on glucose induces the expression of hexokinase 2 (Hxk2 encoded by *HXK2*), activates ethanol-producing metabolism, and represses mitochondrial activities (Moreno and Herrero, 2002). In contrast, this glucose-dependent repression on the respiratory energy metabolism can be dysregulated by xylose (Jin et al., 2004). The mitochondrial genes and the TCA cycle are very active when *Sa. cerevisiae* are grown on xylose. In addition, xylose can upregulate the glyoxylate pathway and activate cytosolic acetyl-CoA metabolism with enhanced expression of genes encoding aldehyde dehydrogenases and acetyl-CoA synthetase 1 (*ALD2*, *ALD3*, *ALD6*, and *ACS1*). Meantime, the gene encoding ethanol reoxidizing alcohol dehydrogenase (ADH2) is also highly induced upon xylose utilization (Matsushika et al., 2014). Besides, the non-oxidative PPP is activated when recombinant *Sa. cerevisiae* is grown on xylose, leading to the accumulation of some important intermediates such as E-4-P, which is one of the precursors for the synthesis of aromatic compounds (Figure 2). All of these metabolic properties make *Sa. cerevisiae* beneficial for the biosynthesis of many classes of natural products, such as terpenoids, flavonoids, and other polyphenols.

To direct the production of natural compounds from xylose, the relative pathways need to be constructed and introduced into xylose-utilizing *Sa. cerevisiae*. Currently, production of vitamin A, protopanaxadiol, *p*-coumaric acid, carotenoid, and other natural products has been achieved in *Sa. cerevisiae* by fermentation on xylose (Table 1; Kwak et al., 2017; Borja et al., 2019; Su et al., 2020). For instance, a lycopene biosynthetic pathway consisting of *CrtE*, *CrtB*, and *CrtI* was introduced into xylose-fermenting *Sa. cerevisiae* overexpressing native *XK* and *Sc. stipitis*-derived *XYL1* and *XYL2*. The PK pathway consisting of xylulose-5-phosphate phosphoketolase (xPK) and phosphotransacetylase (PTA) was further introduced to directly convert xylulose-5-phosphate into acetyl-CoA. The resultant strain produced 1.6-fold more lycopene using the mixture of glucose and xylose than using glucose alone (Su et al., 2020). In another case, squalene-producing recombinant *Sa. cerevisiae* showed 8-fold higher production on xylose than on glucose (Kwak et al., 2017). A recombinant strain of xylose-metabolizing *Sa. cerevisiae* was engineered to carry the pathway for *p*-coumaric acid production through the expression of tyrosine ammonia lyase (TAL) and overexpression of some tyrosine biosynthetic pathway genes (Borja et al., 2019). This strain produced 242 mg/L of *p*-coumaric acid from xylose while the titer was only 5.35 mg/L on glucose. Moreover, a xylose-fermenting strain expressing the biosynthetic pathway of shinorine, a natural sunscreen material, produced a trace amount of shinorine in glucose, whereas the titer was dramatically increased by adding xylose in the medium (Park et al., 2019). This interesting result was related to enhanced PPP flux triggered by xylose and abundant supply of sedoheptulose-7-phosphate, which is the preliminary precursor for the synthesis of shinorine.

**Table 1:** Summary of the biosynthesis of natural products from xylose using yeast strains.

Chassis	Engineering strategy	Product	Titer (mg/L)	References
*Scheffersomyces stipitis*	↑DAHP synthase variant (aro4K220L), ↑ aro1(D900A), and ↑ Tkt1	Shikimate	3,110	Gao et al., 2017
*Saccharomyces cerevisiae*	↑ SsXYL1, ↑SsXYL2, ↑SsXYL3, ΔARO10, ΔPDC5, ↑shikimate kinase II (aroL) from *E. coli*, ↑tyrosine ammonia-lyase (TAL) from *Flavobacterium johnsoniae*, ↑DAHP synthase mutant, ↑chorismate mutase mutant	*p*-coumaric acid	242	Borja et al., 2019
*Saccharomyces cerevisiae*	↑ SsXYL1(K271N), ↑ SsXY2, ↑XKS,↑Gal2(N376F), ↑xPk,↑PTA, ↑tHMG1, ↑CrtEBI, Δpho13, and ΔAld6	β-Carotene	903	Su et al., 2020
*Saccharomyces cerevisiae*	↑ SsXYL1, ↑SsXYL2, ↑SsXYL3, ↑TKL1, ↑NpR5600, ↑NpR5599, ↑NpR5598, ↑NpR5597, and ΔTAL1	Shinorine	31	Park et al., 2019
*Saccharomyces cerevisiae*	↑ SsXYL1, ↑SsXYL2, ↑SsXYL3, ↑TKL1, ↑ERG10, ↑tHMG1, ↑TAL1, ΔTAL1, Δpho13, and ΔAld6	Squalene	532	Kwak et al., 2017
*Saccharomyces cerevisiae*	↑ SsXYL1, ↑SsXYL2, ↑SsXYL3, ↑TKL1, ↑ERG10, ↑tHMG1, ↑TAL1, ↑ADS, ΔTAL1, Δpho13, and ΔAld6	Amorphadinene	254	Kwak et al., 2017
*Saccharomyces cerevisiae*	↑ SsXYL1, ↑SsXYL2, ↑SsXYL3, ↑CrtE/I/YB, ↑Blh, Δpho13, and ΔAld6	Vitamin A	3,350	Sun et al., 2019
*Yarrowia lipolytica*	↑ XYL1, ↑XYL2, ↑XKS, ↑TAL (tyrosine ammonia lyase), ↑4CL, ↑CHS, ↑CHI, and ↑XT (xylose transporter, YALI0B00396)	Naringenin	715	Wei et al., 2020b

## Biosynthesis of Natural Products From Xylose by *Yarrowia Lipolytica*

*Yarrowia lipolytica* is an obligate aerobe that has a high flux of the TCA cycle and high translational efficiency of mitochondrial genes involved in aerobic respiration (Man and Pilpel, 2007; Christen and Sauer, 2011; Zhu and Jackson, 2015; Abdel-Mawgoud et al., 2018; Shi et al., 2018; Ma et al., 2019). This yeast grows at temperatures below 34°C and over a wide pH range, with metabolic performances varying with cultivation conditions (Egermeier et al., 2017; Abdel-Mawgoud et al., 2018). It is a GRAS microbe due to its low tendency of growing at human body temperature and the low probability of causing only mild infections in immunocompromised people (Groenewald et al., 2014). Phylogenetically dissimilar to other members in the yeast family, *Y. lipolytica* is considered to be nonconventional (Dujon et al., 2004). Its genome is naturally in haploid form facilitating genetic manipulation, although diploids are occasionally observed (Knutsen et al., 2007; Abdel-Mawgoud et al., 2018). The gene density in this microbe is much lower compared with *Sa. cerevisiae*, with the large and abundant intergenic regions suitable for gene integration (Dujon et al., 2004; Abdel-Mawgoud et al., 2018; Holkenbrink et al., 2018). The genes in *Y. lipolytica* are rich in introns (Stajich et al., 2007; Mekouar et al., 2010), the presence of which can positively affect expression levels of the relative exons (Le Hir et al., 2003; Hong et al., 2012; Tai and Stephanopoulos, 2013; Shaul, 2017). In addition, this strain has a broad spectrum of carbon sources, including sugars, acetate, fatty acids, alcohols, waste cooking oil, and so on (Abdel-Mawgoud et al., 2018).

Genetic engineering of *Y. lipolytica* is generally challenging compared with *Sa. cerevisiae*. First, it is not easy to precisely integrate gene fragments into the genome of *Y. lipolytica* at specified loci because this organism prefers NHEJ (Richard et al., 2005), whereas homologous recombination usually occurs only when long homologous arms (>1 kb) are used (Verbeke et al., 2013). This is attributed to the Ku70/Ku80 protein heterodimer that repairs breaks in DNA double strands (Lustig, 1999). Deletion of the relevant genes results in much higher frequency in the occurrence of homologous recombination with short (50 bp) homologous arms (Verbeke et al., 2013), and repression of both *KU70* and *KU80* by CRISPR-dCas9 greatly improves the efficiency of homologous recombination (>90%; Schwartz et al., 2017a). Second, there are not many engineering tools available for *Y. lipolytica* given that this yeast has only been used in metabolic engineering for less than two decades. The emergence of *Y. lipolytica*-based CRISPR systems (Gao et al., 2016; Schwartz et al., 2016, 2017b, 2018; Morse et al., 2018; Zhang et al., 2018), transposon systems (Casaregola et al., 2000; Patterson et al., 2018; Wagner et al., 2018; Yu et al., 2018) and artificial genomes (Guo et al., 2020) has greatly facilitated genetic modifications, although delicate design is always required. A detailed description of all the genetic tools and strategies suitable for *Y. lipolytica* engineering can be found in a very recent review (Ma et al., 2020).

It had long been thought that *Y. lipolytica* could not naturally utilize xylose as the only carbon source (Blazeck et al., 2014; Zhao et al., 2015), and early attempts enabling xylose metabolism relied on introduction of heterologous pathways from S. stipitis, although such a phenotype tends to be unstable and needs to be strengthened through further adaptation for higher expression levels of XR-encoding genes (Ledesma-Amaro et al., 2016; Wu et al., 2019). However, recent studies have discovered a functional endogenous xylose-metabolizing pathway, which can be highly efficient after facile engineering. Wildtype strain PO1f (ATCC MYA-2613) carries genes encoding active XR, XDH, and XKS at low expression levels, which can be upregulated when xylose is used as the sole carbon source (Ryu et al., 2015; Rodriguez et al., 2016), although it is reported that the two XR-encoding genes are constitutively expressed at stable levels irrespective of the growth stage or the carbon source used for cell cultivation (Rodriguez et al., 2016). Overexpression of XDH- or XKS-encoding gene alone or in combination considerably improves xylose assimilation and conversion (Ryu et al., 2015; Rodriguez et al., 2016), whereas simultaneous overexpression of XR and XKS does not enable cells to grow on xylose (Wu et al., 2019), suggesting expression of XDH and XKS is the bottleneck in natural xylose metabolism (Ryu et al., 2015; Rodriguez et al., 2016). In addition, strain PO1f contains five putative xylose-specific transporters (Ryu et al., 2015), and overexpression of the transporter YALI0B00396 (a co-transporter for xylose and cellobiose) improves xylose uptake (Ryu and Trinh, 2018; Wu et al., 2019; Wei et al., 2020b). Interestingly, xylose metabolism in strain PO1f is not repressed by the presence of glucose (Ryu et al., 2015) as long as glucose concentration is below 2 g/L (Rodriguez et al., 2016), which is distinct from another natural xylose-utilizing strain PO1g whose xylose metabolism is mildly repressed by glucose (Tsigie et al., 2011). These studies have opened up an avenue to xylose utilization in *Y. lipolytica* without the need of complicated genetic engineering, despite the fact that xylose-based cell growth is, in many cases, slower compared with cell growth on glucose.

*Yarrowia lipolytica* has excellent capability of accumulating acetyl-CoA and malonyl-CoA, and is hence a theoretically preferred host for the production of fatty acids, terpenoids, flavonoids, and other compounds that use acetyl/malonyl-CoA as a precursor (Abdel-Mawgoud et al., 2018). In the past few years, the potential of this host has been greatly explored in the biosynthesis of useful compounds such as lipids and biofuels from various carbon sources (Zhu and Jackson, 2015; Du et al., 2016; Ledesma-Amaro et al., 2016; Lv et al., 2019; Ma et al., 2019; Palmer et al., 2020; Shang et al., 2020); however, xylose-based biosynthesis of natural products has been scarcely investigated despite attempts to understand and improve xylose metabolism in *Y. lipolytica* (Tsigie et al., 2011; Ryu et al., 2015; Rodriguez et al., 2016; Wei et al., 2020b). On one hand, this is attributed to the lower growth rate and biomass accumulation when cells are cultivated in xylose than in glucose (Ledesma-Amaro et al., 2016); on the other hand, the functional and highly efficient expression of heterologous metabolic pathways relevant to natural product biosynthesis depends on sophisticated tools for genetic manipulation, which are still very limited. Recently, strain ATCC 201249 was engineered to produce the isoprenoid compound protopanaxadiol from xylose (Wu et al., 2019). Overexpression of XR (with K270R/N272D mutations to convert cofactor preference from NADPH to NADH) and XDH from *Sc. stipitis* and endogenous XKS, followed by xylose adaptation and overexpression of xylose transporter YALI0B00396, enabled the strain to consume 20 g/L of xylose in 72 h, reaching an OD_600_ of 32 in shake flasks. Introduction of the biosynthetic pathway of protopanaxadiol together with fusion expression of pathway enzymes and overexpression of genes involved in precursor supply resulted in a titer of 300 mg/L using fed-batch fermentation. In this process, xylose was preferred to glucose as the carbon source, due to the fact that glucose supported fast cell growth and led to rapid generation and accumulation of acetyl-CoA, which was then channeled for the generation of metabolites other than precursors (Wu et al., 2019). In the latest study, a xylose-inducible machinery was designed to couple endogenous xylose utilization and naringenin biosynthesis, producing 511 mg/L of naringenin in 120 h from 20 g/L glucose and 20 g/L xylose in shake flasks without externally added precursors; the titer was further increased to 715 mg/L in 144 h with an elevated xylose concentration (60 g/L; Wei et al., 2020a,b).

Recently, *Y. lipolytica* has been engineered to produce triglycerides, which can form oil droplets inside cells. These droplets are capable of trapping and storing certain heterologously generated compounds, whose overproduction imposes a threat to cell growth. Thus, *Y. lipolytica* can be used as a good host to produce hydrophobic natural products, including terpenoids and aromatics, which are prone to formation of crystals in the cytosol that are harmful to the host. Concomitant generation of triglycerides allows hydrophobic natural compounds to be stored in oil droplets, and hence protects the host and guarantees continuous production of the target compounds.

## Concluding Remarks

Biorefinery of lignocellulosic hydrolysates as feedstocks to produce advanced fuels and chemicals holds great promise to develop sustainable bioeconomy. As the second most abundant monosaccharide in lignocellulosic hydrolysates, xylose has been attracting much attention for its efficient utilization and conversion into valuable compounds beyond ethanol. Metabolic engineering of yeasts to convert xylose into natural products is a promising option to implement the biotransformation due to some special characteristics of certain yeast strains, such as moderate tolerance to toxic inhibitors in lignocellulosic hydrolysates, and moderate compatibility of heterologous pathway genes derived from both prokaryotes and eukaryotes. Recently, metabolic engineering of *Sc. stipitis*, *Sa. cerevisiae* and *Y. lipolytica* has been accomplished for the biosynthesis of natural products, mainly terpenoids, flavonoids, and aromatics. Some of these microbial production processes function better on xylose than on glucose, as xylose induces reprograming of central metabolism and enhances the supply of key precursors, such as acetyl-CoA.

Currently, most of the studies are centered on *Sa. cerevisiae* due to the availability of facile genetic engineering tools and methods. However, this yeast relies on heterologous pathways for xylose utilization, which are not always highly efficient due to cofactor imbalance. *Sc. stipitis* is a superior xylose-utilizer and, when grown on xylose, generates many intermediate metabolites that are critical for the formation of natural compounds. Nevertheless, the lack of physiological information and genetic engineering tools limits its applications mainly to ethanol production. It appears that *Y. lipolytica* has the merits of both *Sa. cerevisiae* and *Sc. stipitis*. This organism contains native xylose-utilizing genes, and can be turned into an efficient xylose-utilizing factory upon overexpression of these native genes. In addition, the gene arrangement in its genome as well as the established gene manipulation strategies allows for the construction of *Y. lipolytica* workhorse for the generation of natural compounds from xylose. However, the growth rate of this yeast is generally much lower on xylose than on glucose, making the whole bioconversion inefficient. Extensive engineering work is needed to overcome these difficulties.

Another challenge associated with the utilization of lignocellulosic hydrolysates is the presence of toxic compounds, such as furfural and phenolics, generated in the physiochemical pretreatment of biomass. These compounds greatly suppress the growth of fermentation microorganisms and inhibit the biosynthesis of the target products. Thus, development of highly tolerant yeast strains is of critical significance. Strain adaptation or engineering should be considered for the construction of highly efficient yeast cell factories capable of utilizing lignocellulosic hydrolysates for the generation of natural products.

## Author Contributions

JZ, WQ, and XW conceived the project. JZ, MY, and XW wrote the manuscript. All the authors read and approved the manuscript.

### Conflict of Interest

The authors declare that the research was conducted in the absence of any commercial or financial relationships that could be construed as a potential conflict of interest.
